# 

**Published:** 1841-04

**Authors:** 


### BOOKS RECEIVED FOR REVIEW.

ENGLISH.

1. On Diseases of the Hip-joint ; with observations on affections of the joints in the puerperal state. With plates. By William Coulson, Surgeon to the Magdalen Hospital, &c. Second Edition, with alterations and additions.—London, 1841. 8vo, pp. 211.

2. Practical Hints on the Cure of Squinting, by operation. By F. W. Grant Calder, Assistant Surgeon to the Second Regiment of Life Guards.—Lond. 1841. 8vo, pp. 96.

3. Memoranda regarding the Royal Lunatic Asylum, Infirmary, and Dispensary, of Montrose ; with observations on some other institutions of a like nature, and an appendix of documents, partly relating to restraint in the treatment of insanity. By

R. Poole, M.D. Superintendant of the Montrose Asylum.—Mont. 1841. 8vo, pp. 278.

4. Malta considered with reference to its eligibility as a Place of Residence for Invalids. Addressed to the members of the medical profession. By Francis Sankey, M.D. Universitatis Melitensis.—*No date.* 8vo, pp. 23.

5. A Second Appeal to the People of Pennsylvania on the subject of an Asylum for the Insane Poor of the Commonwealth. —Philadelphia, 1840. 8vo, pp. 35.

6. The Retrospect of Practical Medicine and Surgery, No. II. By W. Braithwaite, Surgeon.—London, 1840. 4s. 6d.

7. Medical Reform, in a Letter to Lord Melbourne. By Martin Sinclair, M.D.—London, 1841. 8vo, pp. 36.

8. The Anatomy of the Nerves of the Uterus. By Robert Lee, M.D. F.R.S. With two plates.—London, 1841. Folio, pp. 8.

9. Essays and Heads of Lectures on Anatomy, Physiology, Pathology, and Surgery. By the late Alex. Monro Secundus, M.D. F.R.S.E. &c. upwards of fifty years Professor of Anatomy and Surgery in the University of Edinburgh, with a Memoir of his Life, and copious notes, &c. by his Son and successor. With engravings.—Edinb. 1840. 8vo, pp. 292.

10. The Cause and Treatment of Curvature of the Spine, and Diseases of the Vertebral Column. Illustrated with cases and plates. By E. W. Tuson, F.R.S. F.L.S. Surgeon to the Middlesex Hospital.—London, 1841. 8vo, pp. 283. 10s. 6d.

11. The Domestic Management of the Sick Room, necessary, in aid of Medical Treatment for the Cure of Diseases. By A. T. Thomson, M.D. F.L.S. &c.—London, 1841. 8vo, pp. 506. 10s. 6d.

12. Elements of Physiology for the use of Students, and with especial reference to the wants of Practitioners. By Rudolph Wagner, M.D. Professor of Anatomy in the University of Gottingen. Translated from the German, with additions, by Robert Willis, M.D. &c. Part I. on Generation. —London, 1841. 8vo, pp. 229. 10s. 6d.

13. The Library of Medicine. Arranged and edited by Alexander Tweedie, M.D. F.R.S. Vol. VI. A System of Midwifery. By Edward Rigby, M.D.—London, 1841. 8vo, pp. 314. 10s. 6d.

14. The Mineral Springs of England, and their Curative Efficacy: with remarks on bathing, and on artificial mineral waters. By Edwin Lee, Esq. M.R.C.S.—London, 1841. 12mo, pp. 124. 3s. 6d.

15. Seventh Annual Report of the Trustees of the State Lunatic Hospital at Worcester (U. S.).—Bost. 1840. 8vo, pp. 102.

16. The American Medical Almanack for 1841, designed for the daily use of practising Physicians, Surgeons, Students, and Apothecaries, &c. By J. V. C. Smith, M.D. Vol. III. Continued annually. 18mo, pp. 148.

17. A few Hints addressed to Medical Students about to visit the Parisian Hospitals. By a Physician.—London, 1841. 12mo, pp. 56. 1s. 6d.

18. Annual Report by the Managers of the Royal Edinburgh Lunatic Asylum for the year 1840.—Edinb. 1841. 8vo, pp. 14.

19. The Surgical Anatomy of Inguinal Hernia, the Testis and its coverings. By Thos. Morton, Demonstrator of Anatomy in University College.—London, 1841. Royal 8vo, pp. 330. 9s. Illustrated with lithographic plates and wood engravings, 12s.

20. A Practical Treatise on the Venereal Disease. Founded on lectures delivered at the Aldersgate School of Medicine. By F. C. Skey, F.R.S. Assistant Surgeon of St. Bartholomew's Hospital. With plates. —London, 1841. Sm. 8vo, pp. 195. 4s. 6d.

21. Observations on the Management of Madhouses, Part the Second. By Caleb Crowther, M.D.—London, 1841. 12mo, pp. 104. 2s. 6d.

22. A Series of Anatomical Sketches and Diagrams. Part IV. By T. Wormald and A. M. M'Whinnie, Esq.—London, 1841.

23. A Practical Treatise on the Diseases of the Liver and Biliary Passages. By W. Thomson, M.D. one of the Physicians of the Royal Infirmary of Edinburgh.—Edinb. 1841. 8vo, pp. 306. 8s.

24. A Treatise on the Physiological and Moral Management of Infancy. By Andrew Combe, M.D. Second edition.—Edinb. 1841. 8vo, pp. 380. 6s.

25. Statistical Researches relative to the Etiology of Pulmonary and Rheumatic Diseases. By S. Forry, M.D. &c.—Philadelphia, 1840. 8vo, pp. 44.

26. Medical Communications of the Massachusett's Medical Society. Vol. VI. Part IV.—Boston, 1840.

27. The Principles and Practice of Obstetric Medicine and Surgery, in reference to the process of Parturition. With 100 illustrations on steel and wood. By F. H. Ramsbotham, M.D.—London, 1841. 8vo, pp. 672. 1*l.* 2s.

28. New Remedies: the method of preparing and administering them, &c. Third edition, with numerous modifications and additions. By R. Dunglison, M.D. Sec. A.P.S.—Philadelphia, 1841. 8vo, pp. 541.

29. The Anatomy of the Arteries of the Human Body, with an atlas of plates. By Richard Quain, and Joseph Maclise, Esq. Parts IV. and V.—London, 1841. 12s.

### INDEX TO VOL. XI.

OF THE

### BRITISH AND FOREIGN MEDICAL REVIEW.

| | PAGE |
|---|---|
| Acephalocysts in the brain . | 517 |
| Africa, climate and diseases of . | 365 |
| Air-cells, morbid anatomy of . | 405 |
| Air-passages, foreign body in . | 530 |
| Alison, Dr. on instinct . . | 90 |
| on the poor of Scotland | 1 |
| Alumina in ulcerated bowels . | 524 |
| Alcock, Mr. on injuries of the joints | 459 |
| Amaurosis . . . | 75 |
| Ammon, Dr. von, on strabismus . | 474 |
| Amenorrhœa, Dr. Ashwell on . | 178 |
| Amputation, statistics of . | 256 |
| Anatomy, morbid, Dr. Hodgkin's | 401 |
| demonstrations of . | 220 |
| Andral on the state of the blood | 242 |
| Aneurism, Mr. Thomson on . | 463 |
| Angina pectoris, fatal case of . | 238 |
| Animal heat, observations on . | 393 |
| Arnold, Dr. on the nerves . | 517 |
| Antimony, on poisoning by . | 37 |
| Apoplexy from fright . . | 235 |
| Army, medical statistics of the . | 365 |
| Arnott, Mr. his cause of tumour . | 452 |
| Arteries, Mr. Quain's anatomy of | 210 |
| Arsenic, on poisoning by . | 37 |
| Ascites, on compression in . | 237 |
| Ashwell, Dr. on diseases of women | 174 |
| Asthma, spasmodic, Dr. Graves on | 264 |
| Bell, Sir C. on the roots of the nerves | 278 |
| strabismus . | 474 |
| Bezoar, medical effects of . | 233 |
| Billard, M. on diseases of infants | 198 |
| Births and deaths in England . | 422 |
| Bladder, wound of . . . | 251 |
| Blood, coagulation of, after death | 255 |
| Blood-corpuscles . . | 448 |
| Blood, Dr. Mandl on . . | 225 |
| on the odour of . . | 226 |
| state of in various diseases | 242 |
| Books received for review . | 278-553 |
| Boyle, Mr. on the coast of Africa | 365 |
| Brain, softening of . . | 464 |
| Braithwaite, Mr. his retrospect | 224 |
| Breschet M. on rabies . . | 229 |
| Bronchi, morbid anatomy of . | 402 |
| Bronchocele in the fœtus . | 229 |
| Browne, Dr. on bleeding in mania | 543 |
| Budd, Dr. on pulmonary emphysema | 448 |
| Bull, Dr. on children . . | 224 |
| Burns and Scalds, surgery of . | 311 |
| Burton, Dr. on the gums . | 450 |
| Bushnan, Dr. on instinct . | 90 |
| Cæsarean operation, successful | 530 |
| Calculous diseases, Dr. Prout on | 330 |
| Calculus, sulphur in cystic oxide | 458 |
| Calder, Mr. on strabismus . | 474 |
| Callus, absorption of . . | 353 |
| Cancer, Dr. Walshe on . . | 158 |
| Cape of Good Hope, diseases of . | 377 |
| Carbon, assimilation of, by plants | 437 |
| Carotid artery, rupture of . | 338 |
| Carotid, ligature of . . . . | 528 |
| Carswell, Dr. testimonial to . | 269 |
| Cartilage, vascularity of . . | 451 |
| Cataract . . . | 79-86 |
| Catheter, new mode of introducing | 535 |
| Chelius, his surgery . . | 305 |
| Chemical philosophy, recent progress of | 130 |
| Chemistry, Dr. Kane's elements of | 514 |
| organic . . | 436 |
| treatises on . . | 129 |
| Chest, on diseases of the . | 217 |
| Children, on the diseases of . | 198 |
| on the management of | 224 |
| Chlorosis . . . . | 175 |
| Cholesterine in morbid fluids . | 254 |
| Chomel, M. his lectures . | 516 |
| Chorea, treated by ammon. of copper | 235 |
| Chromic acid; microscopical use of | 520 |
| Ciliary motions . . . | 521 |
| Circulation, Valentin on the . | 300 |
| Cleland Dr. on tobacco . . | 512 |
| Climate of Africa . . | 366 |
| Climate of England, effects of . | 430 |
| Club-foot, Dr. Mütter on . | 513 |
| Cold, effects of . . . | 315 |
| Commentaries, Dr. Paine's . | 383 |
| Compression in ascites . . | 237 |
| Concretion, stony, on the nose . | 238 |
| Congestion, venous . . | 398 |
| Conjunctiva, anatomy of . | 59 |
| diseases of . . | 60 |
| Conolly, Dr. his report on insanity | 104 |
| on insanity among the poor | 271 |
| Consumption, mortality in England | 425 |
| Contagion, theory of action of . | 442 |
| Convulsions, case of peculiar . | 234 |
| Cooper, Sir A. obituary notice of | 549 |
| his surgery . | 305 |
| Copaiba, action of . . | 529 |
| Copper, naturally in the body . | 226 |

| | PAGE. |
|---|---|
| Copper, on poisoning by . | 37 |
| Cornea, diseases of . . | 66 |
| Cowan, Dr. his statistics of Glasgow | 1 |
| Cows, on the gestation of . | 273 |
| Croup . . . . . | 206 |
| tracheotomy successful in | 526 |
| Crowther, Dr. on madhouses . | 515 |
| Cubebs, action of . . | 529 |
| Dalrymple, Mr. on the organization of lymph . . . | 458 |
| Daniell, Mr. his work on chemistry | 129 |
| Daubeny, Dr. on the atomic theory | 129 |
| Davis, Dr. on hydrocephalus . | 151 |
| Death, causes of, in England . | 428 |
| Diabetes, use of tinct. mur. ferri in | 265 |
| Dick, Dr. on deranged digestion . | 223 |
| Dieffenbach, Dr. his surgery . | 194 |
| Digestion, Valentin on . . | 300 |
| philosophy of . . | 397 |
| on the derangements of | 223 |
| Digitalis in epilepsy . . | 509 |
| Duchatelet, M. on hygiene . | 1 |
| Duffin, Dr. on strabismus . | 474 |
| Electro-metallurgy, treatise on . | 510 |
| Elliott, Mr. on strabismus . | 474 |
| Ellis, Mr. his anatomy . . | 220 |
| Elliotson, Dr. his physiology . | 497 |
| Emphysema, pulmonary . . | 448 |
| Entropium and ectropium . | 75 |
| Epidermis, exfoliation of . | 200 |
| Epilepsy, digitalis in . . | 509 |
| Erysipelas, treatment of . . | 321 |
| Evanson, Dr. on diseases of children | 198 |
| Eye, on the action of . . | 521 |
| Dr. Hull, on diseases of . | 221 |
| Mr. Tyrrell on diseases of the | 55 |
| Farr, Mr. on the causes of death, | 423 |
| Fauces, morbid anatomy of . | 411 |
| Fever, on the causes of typhus . | 1 |
| on the coast of Africa . | 368 |
| Filth and fetor, alleged causes of fever | 6 |
| not operative in Paris | 14 |
| Fissure of the anus, treatment of | 251 |
| Fistula treated by boiling water | 252 |
| Fœtus, on the diseases of . | 212 |
| Fracture of the neck of the femur | 262 |
| Fuchs, Dr. on softening of the brain | 464 |
| Funis, on the separation of . | 201 |
| Fistula, cæcal, in the loins . | 248 |
| Gall-bladder, muscularity of . | 524 |
| Galvanism curing paralysis . | 524 |
| Ganglia, on the formation of . | 299 |
| Generation, physiology of . | 300 |
| Geology, elements of . . | 224 |
| Gestation of cows, period of . | 273 |
| Glanders artificially produced . | 230 |
| Glands of the throat, disease of . | 239 |
| Glasgow, vital statistics of . | 1 |
| Gonorrhœa, chloride of zinc in . | 527 |
| Gout cured by animal electricity . | 233 |
| Graham, Mr. his chemistry . | 223 |
| Graves, Dr. on asthma . . | 264 |
| Guerin, M. on the action of muscles | 250 |

| | PAGE. |
|---|---|
| Gulliver, Mr. on blood-corpuscles | 448 |
| Haeser, Dr. his researches . | 515 |
| Hall, Mr. on strabismus . . | 474 |
| Dr. M. on the vis nervosa . | 522 |
| on the pathology of the nerves | 452 |
| Hanwell asylum . . | 271 |
| on seclusion at . . | 546 |
| Hawkins, Mr. on bodies in the larynx | 452 |
| Headach, treatment of . . | 235 |
| Hearing, on the organs of . | 222 |
| Heart, on the white spots of . | 448 |
| Heart and vessels, fatal diseases of | 426 |
| Hemorrhage, transfusion in . | 260 |
| Hernia, radical cure of . . | 258 |
| case of . . . | 448 |
| Hodgkin, Dr. his morbid anatomy | 401 |
| Houston, Dr. his catalogue . | 513 |
| Horse, on the foot of the . . | 224 |
| Hull, Dr. on diseases of the eye . | 231 |
| Humoral pathology, on the . | 392 |
| Hydrocephalus, acute, treatise on | 151 |
| Hydrogen, its assimilation in plants | 439 |
| Hydrophobia, observations on . | 437 |
| Induration, infantile . . | 202 |
| Infants, on the diseases of . | 198 |
| Inflammation, theories of . | 398 |
| Insanity, treatises on . . | 104 |
| Instinct, treatises on . . | 90 |
| Intestines, morbid anatomy of . | 412 |
| Iris, inflammation of . . | 71 |
| Irrigation, fetid, evils of . . | 1 |
| Itch, treatment of . . | 246 |
| Johnstone, Dr. on sensation . | 505 |
| Joints, on the injuries of . | 455 |
| dropsy of, treated by antimony | 247 |
| Kane, Dr. his chemistry . | 514 |
| Kidneys, enlargement of . | 227 |
| Knee-joints, removal of bodies from | 526 |
| Kyanised timber, danger of . | 532 |
| Labour, on tumours impeding . | 500 |
| Labouring classes, on the health of | 1 |
| Lacrymal apparatus, diseases of . | 76 |
| Lallemand, M. on zoospermata . | 520 |
| Larynx, foreign bodies in . | 452 |
| Lead, naturally in the body . | 226 |
| Lead, its effect on the gums . | 450 |
| Lee, Dr. his geology . . | 224 |
| on the nerves of the uterus | 493 |
| Lens, inflammation of . . | 79 |
| Liebig, M. his organic chemistry | 436 |
| Life, Dr. Paine on . . | 385 |
| Liston, Mr. on secreting surfaces | 451 |
| his surgery . . | 305 |
| Lithotrity, origin of . . | 270 |
| London, on the fevers of . . | 5 |
| bills of mortality of . | 545 |
| Lotions, ophthalmic . . | 57 |
| Loxarthus, treatise on . . | 513 |
| Lucas, Mr. on strabismus . | 474 |
| Lunatics, treatment of . . | 546 |
| Lungs, tuberculous disease of . | 407 |
| Lymph, on the rapid organization of | 458 |
| Lymphadenitis, idiopathic, case of | 241 |

| | PAGE |
|---|---|
| Madhouses, management of . | 515 |
| Mania, on bleeding in . . | 543 |
| Mandl, Dr. on the blood . | 225 |
| Markham, Dr. on Parisian surgery | 218 |
| Mauritius, diseases of . . | 379 |
| Maunsell, Dr. on diseases of children, | 195 |
| Medicine, retrospect of . . | 224 |
| Medico-chirurgical transactions . | 447 |
| Medulla oblongata, anatomy of . | 524 |
| Melchior, M. on strabismus . | 474 |
| Menstruation in a child . | 225 |
| vicarious . | 179 |
| Mercer, Dr. on the organs of hearing, | 222 |
| Mercurial ointment, new mode of preparing . . . | 254 |
| Milk, microscopic contents of . | 228 |
| Mortality of London, tables of | 267-545 |
| England and Wales, | 422 |
| Motion, Valentin on . . | 300 |
| Mucous membranes, morbid anat. of, | 401 |
| Milligen, Dr. on insanity . | 103 |
| Muscles, extensive section of . | 250 |
| Museum, Dublin, catalogue of . | 513 |
| Naegele, Dr. on contracted pelvis, | 167 |
| Nasse, Dr. on milk . . | 228 |
| Navy, statistical reports of . | 182 |
| Nerve, olfactory, functions of . | 279 |
| optic, functions of . | 280 |
| oculo-motor, function of | 282 |
| pathetic, function of . | 283 |
| abductor, function of . | 283 |
| facial, function of . | 286 |
| glosso-pharyngeal, functions of | 287 |
| hypo-glossal . . | 290 |
| par vagum . . | 291 |
| spinal accessory . . | 294 |
| phrenic . . . | 294 |
| sympathetic . . | 293 |
| Nerves, 5th pair, functions of . | 284 |
| pneumogastric, experiments on | 517 |
| primitive fibres of . | 299 |
| on the functions of . | 277 |
| Nervous diseases, mortality from | 424 |
| system, pathology of | 452 |
| Nose, stony concretion on . | 238 |
| Nutrition, Valentin on . . | 301 |
| Œsophagus, morbid anatomy of . | 412 |
| Ophthalmia, varieties of . . | 60-64 |
| Orfila, M. on poisoning . | 37 |
| Ovaritis from injections . | 246 |
| Over-eating, effects of . . | 232 |
| Paget, Mr. on the spots of the heart | 448 |
| Paine, Dr. his physiology . | 383 |
| Palate, fissure of . . | 196 |
| Paris, on the surgical practice of | 218 |
| Paralysis cured by galvanism . | 524 |
| Pelvis, case of tumour in . . | 531 |
| Pelvis, contracted . . | 167 |
| on tumours in . . | 500 |
| Percival, Mr. his narrative . | 104 |
| Peritonitis, new signs of . | 234 |
| Perineum, rupture of . . | 195 |
| Physiology, Dr. Elliotson's . | 497 |
| Physiology, Wagner's elements of | 507 |
| Placenta, structure of . . | 540 |
| Plastic operations, Dieffenbach's | 197 |
| Placenta, on the structure of . | 459 |
| Platina, medicinal use of . | 523 |
| Pneumonia, morbid anatomy of | 406 |
| lectures on . . | 516 |
| Poisoning, M. Orfila on . | 37 |
| Prolapsus uteri, new treatment of | 197 |
| Prout, Dr. on calculous diseases | 330 |
| Puchelt on pelvic tumours . | 500 |
| Pupil artificial . . . | 87 |
| Quain, Mr. on the arteries . | 210 |
| Rabies, on the transmission of . | 229 |
| Ray, electric, a cure for gout . | 233 |
| Reflex function . . | 300 |
| Registrar-general's report . | 422 |
| Reid, Dr. on the placenta . | 540 |
| Respiration, Valentin on . | 300 |
| Respiratory organs, diseases of . | 425 |
| Restraint in lunatic asylums . | 105 |
| Rhatany, in anal fissure . | 251 |
| Scarlatina, on dropsy after . | 240 |
| Sclerotica, anatomy of . . | 69 |
| Scotland, on the poor in . | 1 |
| Seclusion, Dr. Conolly on . | 446 |
| Senegal, diseases of . . | 365 |
| Sensation, Dr. Johnstone on . | 505 |
| Sick room, domestic management of | 511 |
| Skey, Mr. on the venereal disease | 508 |
| Skin, coloration of in infants . | 199 |
| Skull, fractured in parturition . | 253 |
| Sierra Leone, disease of . . | 365 |
| Smallpox, eccoprotic treatment of | 246 |
| mortality from . | 432 |
| law of . | 434 |
| Smee, Mr. on electro-metallurgy | 510 |
| Softening of the brain . . | 464 |
| Spinal cord, case of disease of . | 451 |
| marrow, lesion of . . | 525 |
| Spleen, engorgements of . . | 236 |
| Spooner, Mr. on the horse's foot | 224 |
| St. Helena, diseases of . . | 375 |
| Stafford, Mr. his case of cut throat | 459 |
| Stanley, Mr. on diseases of spinal cord | 451 |
| Stethoscope, Dr. Bird's flexible . | 544 |
| Stomach, morbid anatomy of . | 412 |
| and brain, sympathy of | 506 |
| Strabismus, treatises on . . | 474 |
| causes of . . | 478 |
| morbid anatomy of . | 479 |
| effects of operation for | 489 |
| statistics of . . | ib. |
| accidents from operation for | 491 |
| Surgery, treatises on . . | 305 |
| Swainson, Mr. on instinct . | 90 |
| Teeth, chemistry of tartar of . | 533 |
| Temperance, effects of, in India . | 259 |
| Thevenot, M. on Senegal . | 365 |
| Thomson, Dr. his organic chemistry | 129 |
| on the sick room . | 511 |
| Tobacco, essay on . . | 512 |
| Tonsils, calculus in . . | 528 |

PAGE.

Torticollis, new species of . 250

Tracheotomy, cases of . 452, 526-30

Trigeminus nerve, irritation of . 230

Transfusion in hemorrhage . 260

Travers, Mr. his case of hernia . 448

Tubercles in the larynx . . 239

Tulloch, Major, his army reports 365

Tyrrell, Mr. on diseases of the eye 55

Urinary diseases, mortality from . 427

Dr. Prout on . 330

functions, Valentin on . 300

Urine after scarlatina . . 240

Uterus, on the nerves of . 493

Valentin on the functions of nerves 277

Varicocele, radical cure of . 529

Variola, case of posthumous . 236

PAGE.
Venereal disease, treatises on . 508
Vidal M. on surgery . . 305
Vis nervosa of Haller, Dr. Hall on 522
Volkmann, on the muscles of the eye 521
Wagner, Dr. his physiology . 507
Waller, Dr. on diseases of the womb 174
Walshe, Dr. on cancer . . 158
Water, boiling, in fistula . 252
White swellings, treatment of . 529
Wightman, Dr. on nervous sympathy 506
Williams, Dr. on auscultation . 217
Willis, Dr. his translation of Wagner 507
Wilson, Dr. his navy reports . . 184
Women, on the diseases of . 114
Womb, on diseases of . . 174
Wounds, surgery of . . 306

END OF VOL. XI.

PRINTED BY C. ADLARD, BARTHOLOMEW CLOSE.

